# Linking 23 physical activity intensity levels to health-related quality of life in 10-year-old children

**DOI:** 10.1186/s13104-025-07478-8

**Published:** 2025-10-02

**Authors:** Mari Stai, Eivind Aadland, John Roger Andersen

**Affiliations:** 1 Faculty of Health and Social Sciences, Department of Health and Caring Sciences, Western Norway University of Applied Sciences, Førde, Norway; 2 Department of Sport, Food and Natural Sciences, Faculty of Education, Arts and Sports, Western Norway University of Applied Sciences, Sogndal, Norway; 3 Førde Hospital Trust, Førde, Norway

**Keywords:** Physical activity, Health-related quality of life, Physical well-being, Overweight, Obesity, Children

## Abstract

**Objectives:**

To examine the associations between 23 accelerometer-derived physical activity levels and health-related quality of life (HRQoL) in 10-year-old children.

**Results:**

This study analyzed cross-sectional baseline data from the ASK study, a randomized controlled trial. The Kidscreen-27 questionnaire assessed HRQoL, with the “physical well-being” domain as the primary outcome. Physical activity levels were measured using ActiGraph GT3X + accelerometers, capturing 23 intensity bands ranging from 0 to 99 to ≥ 10,000 counts per minute (cpm). Partial least squares regression analysis was used to explore the associations between physical activity and HRQoL. The study included 722 fifth-grade children (mean age 10.2 ± 0.3 years, mean waist circumference 61.8 ± 7.4 cm, 51% male). In the overall cohort, physical activity accounted for 5.23% of the variance in physical well-being, with 2.03% for girls and 9.06% for boys. Among boys with overweight/obesity, the explained variance increased to 20.03%, compared to 8.63% for those without. For girls, no association was observed for those without overweight/obesity, whereas 8.93% of the variance was explained for those with overweight/obesity. High-intensity physical activity (6000–7000 cpm) showed the strongest associations, though positive relationships were evident across all intensity levels.

**Supplementary Information:**

The online version contains supplementary material available at 10.1186/s13104-025-07478-8.

## Introduction

In children, physical activity is linked to better health-related quality of life (HRQoL), defined as physical, psychological, and social well-being [1], with the most valid empirical data from accelerometer-based studies [2–5]. Although effect sizes are typically small to moderate, they may still be clinically meaningful. Children with lower initial levels of physical activity and HRQoL may be at increased risk for more pronounced negative outcomes over time [6]. Thus, identifying effective strategies to promote physical activity early in life—alongside other key developmental factors—may be important for supporting children’s health trajectories and enhancing their capacity to meet future life challenges.

A limitation of previous studies is the categorization of physical activity into four broad intensity levels: inactive, light, moderate, and vigorous, which may overlook detailed effects on HRQoL [7]. Another issue is that different physical activity intensity levels depend on each other, posing a statistical challenge for regression models [8, 9].

Consequently, there is a gap in understanding how physical activity intensities affect children’s HRQoL. A solution for studying many intensity levels is to apply partial least squares (PLS) regression, which can handle highly correlated variables. This approach was, for example, successfully used to model how 23 different physical activity levels were associated with cardiometabolic health in children [8]. To our knowledge, this has not been applied when HRQoL is the outcome.

In addition, group differences remain a scarcely explored area. Does the effect vary, for example, by sex or weight status? For instance, girls may engage in physical activity for health or weight-related reasons, whereas boys might participate primarily for enjoyment [10–13]. Children with overweight or obesity might experience unique benefits from physical activity, potentially mitigating adverse health effects associated with excess weight [14].

Thus, this study explored the association between 23 physical activity intensity levels and HRQoL in 10-year-old children. As a secondary analysis, stratified analyses for girls and boys without and with overweight or obesity were conducted.

## Materials and methods

### Design and sample

Cross-sectional baseline data from the cluster-randomized controlled trial “Active Smarter Kids (ASK)”, a school based physical activity intervention in Norway [15–17] were used. The ASK study recruited fifth-grade children (aged 10 years). In the initial cohort, 1129 participants from 57 schools met the inclusion criteria. The dataset analyzed in this study included 722 children with valid data for the variables of interest. Detailed information about the study’s methods has been comprehensively documented in other publications [16, 17].

### Human ethics and consent to participate

The study adhered to the ethical guidelines of the Declaration of Helsinki [18], was approved by the Regional Committees for Medical and Health Research Ethics (reference number: 2013/1893), and is registered at www.Clinicaltrials.gov (https://www.clinicaltrials.gov/). The Clinical trial number is NCT02132494 (registration date: 6th May 2014) [15]. Written consent was obtained from each child’s parents, legal guardians, and the relevant school authorities.

### Variables

Participants’ subjective experience of HRQoL was assessed using the Kidscreen-27 questionnaire, which have shown good psychometric properties in the study sample [19]. Physical well-being was chosen as the primary outcome because previous analyses have shown that physical HRQoL was the dimension most strongly linked to total daily physical activity in younger children in this cohort [20]. Kidscreen-27 captures five dimensions of HRQoL: physical well-being, psychological well-being, autonomy and parents, social support and peers, and the school environment. The anticipated population score for these dimensions is characterized by a mean T-score of 50 and a standard deviation of 10, with higher scores indicating better HRQoL [1]. The Kidscreen-27 scale physical well-being encompasses the aggregate of responses to the following five inquiries: “In general, how would you say your health is? Have you felt fit and well? Have you been physically active (e.g., running, climbing, biking)? Have you been able to run well? Have you felt full of energy?“. The physical well-being scale demonstrated a Cronbach’s alpha of 0.80 in the present sample. A floor effect of 0.1% and a ceiling effect of 7.3% were observed. The test-retest reliability was displayed by an intraclass correlation coefficient of 0.73. The confirmatory factor analysis revealed an acceptable overall model fit [19].

Physical activity was recorded using an accelerometer (ActiGraph GT3X+, LLC, Pensacola, Florida, USA) worn at the waist for seven days, except during water activities or sleeping [15]. Accelerometers were initialized at a sampling rate of 30 Hz and analyzed using 1-second epochs using Kinesoft software (version 3.3.80, Kinesoft, Loughborough, UK). After removing consecutive periods of ≥ 60 min of zero counts, children had to have ≥ 8 h of wear time per day for ≥ 4 days (between 06:00 and 23:59) to be included in the analysis. The physical intensity spectrum was described using 23 variables representing different intensity levels, ranging from 0 to 99 to ≥ 10,000 counts per minute (cpm). A variable capturing the sum of all physical activities was included (total cpm). We regarded intensities 0–99 cpm as sedentary time, 100–2295 cpm as light intensity, 2296–4011 cpm as moderate intensity, and ≥ 4012 cpm as vigorous intensity [21]. The number of physical activity intensity-level variables can vary depending on how the intensity spectrum is segmented. In the present analysis, we selected a resolution aimed at capturing meaningful variation across the full range of intensities. Although no standardized approach exists, the use of 23 intensity variables was judged to offer a balanced and interpretable representation of association patterns.

Waist circumference was measured using an ergonomic measuring tape (SECA GmbH, Hamburg, Germany). Based on the age- and sex-adjusted reference values for waist circumference, a variable was created to categorize participants as not having or overweight/obesity [22].

### Statistical analyses

The characteristics of the sample are presented using descriptive statistics. PLS regression was used to explore the multivariate associations between physical well-being and physical activity intensity levels (explanatory variables) [7, 23, 24]. All variables were centered and standardized to unit variance before PLS regression. The number of components in the PLS models was cross-validated using Monte Carlo resampling with 100 repetitions, with random samples of 50% of the children in the calibration and validation datasets, to estimate the predictive performance of each model [25]. The minimum median root mean squared error of prediction was used as a criterion to avoid overfitting. We adjusted for age, sex, and waist circumference by regressing physical well-being on these variables using ordinary multiple regression. We used residuals from these models in the PLS analyses to discern the unique relationship between physical activity intensity levels and well-being. Secondary separate analyses were conducted for girls, boys, and children with normal weight versus overweight/obesity, stratified by sex. Results were reported as multivariate correlation coefficients along with 95% confidence intervals (CIs), which showed the importance of each physical activity intensity variable within the multivariate space and the explained variance of the total model (R^2^) [10]. Although derived from the multivariate space, the multivariate correlation coefficients can be interpreted equivalently to bivariate correlations ranging from − 1 to 1. Multivariate analyses were performed using Sirius version 11.0 (Pattern Recognition Systems AS, Bergen). All other analyses were performed using IBM Statistical Package for the Social Sciences (SPSS) version 28 for Mac.

## Results

The study encompasses 722 children with complete data on all the relevant variables from a total sample of 1129 fifth graders (10.2 ± 0.3 years, waist circumference 61.8 ± 7.4 cm, 51% boys), detailed in Table 1 and the supplementary material (Suppl. Table 1).

**Table 1:** Characteristics of the total sample, boys, and girls

Variables, mean ± sd	Total sample (*n* = 722)	Boys (*n* = 368)	Girls (*n* = 354)
Age (year)	10.2 ± 0.3	10.2 ± 0.3	10.2 ± 0.3
Waist circumference (cm)	61.8 ± 7.4	62.3 ± 7.2	61.4 ± 7.6
Kidscreen-27			
Physical well-being	51.7 ± 9.8	52.7 ± 10.00	50.5 ± 9.5
Psychological well-being	53.3 ± 9.3	53.3 ± 9.7	53.3 ± 8.9
Autonomy & parents	50.7 ± 9.4	50.6 ± 9.8	50.9 ± 9.1
Social support & peers	51.5 ± 9.3	51.6 ± 9.63	51.3 ± 9.0
School environment	54.2 ± 9.6	53.2 ± 10.23	55.3 ± 8.9
Physical activity (minutes per day)			
Counts per minute	708 ± 275	749 ± 299	666 ± 242
Sedentary	597.7 ± 56	593 ± 59	603 ± 53
Ligh physical activity	121.6 ± 22		
Moderate physical activity	36.7 ± 9.4		
Vigorous physical activity	39.3 ± 14.9		
0–99	597 ± 56	593 ± 59	603 ± 53
100–249	18.0 ± 3.1	17.9 ± 3.2	18.1 ± 3.0
250–499	21.1 ± 3.7	21.1 ± 3.8	21.1 ± 3.6
500–999	30.4 ± 5.6	30.7 ± 5.8	30.0 ± 5.4
1000–1499	22.4 ± 4.5	22.9 ± 4.7	21.9 ± 4.3
1500–1999	20.1 ± 4.4	20.7 ± 4.6	19.5 ± 4.1
2000–2499	14.8 ± 3.5	15.4 ± 3.6	14.2 ± 3.2
2500–2999	12.3 ± 3.1	12.9 ± 3.2	11.7 ± 2.8
3000–3499	11.2 ± 3.1	11.9 ± 3.2	10.6 ± 2.7
3500–3999	7.9 ± 2.4	8.4 ± 2.5	7.4 ± 2.1
4000–4449	6.3 ± 2.1	6.8 ± 2.2	5.8 ± 1.7
4500–4999	5.7 ± 1.9	6.2 ± 2.1	5.2 ± 1.6
5000–5499	4.1 ± 1.4	4.4 ± 1.6	3.6 ± 1.2
5500–5999	3.3 ± 1.2	3.7 ± 1.3	3.0 ± 0.9
6000–6499	3.0 ± 1.1	3.4 ± 1.3	2.7 ± 0.9
6500–6999	2.1 ± 0.9	2.4 ± 1.0	1.9 ± 0.6
7000–7499	1.8 ± 0.7	2.0 ± 0.8	1.6 ± 0.5
7500–7999	1.6 ± 0.7	1.8 ± 0.8	1.4 ± 0.5
8000–8499	1.2 ± 0.5	1.3 ± 0.6	1.0 ± 0.4
8500–8999	1,0 ± 0.4	1.0 ± 0.5	0.9 ± 0.3
9000–9499	0.9 ± 0.4	1.0 ± 0.5	0.8 ± 0.3
9500–9999	0.6 ± 0.3	0.7 ± 0.4	0.6 ± 0.3
≥ 10,000	7.7 ± 6.7	8.1 ± 7.4	7.3 ± 5.7

**Fig. 1:**
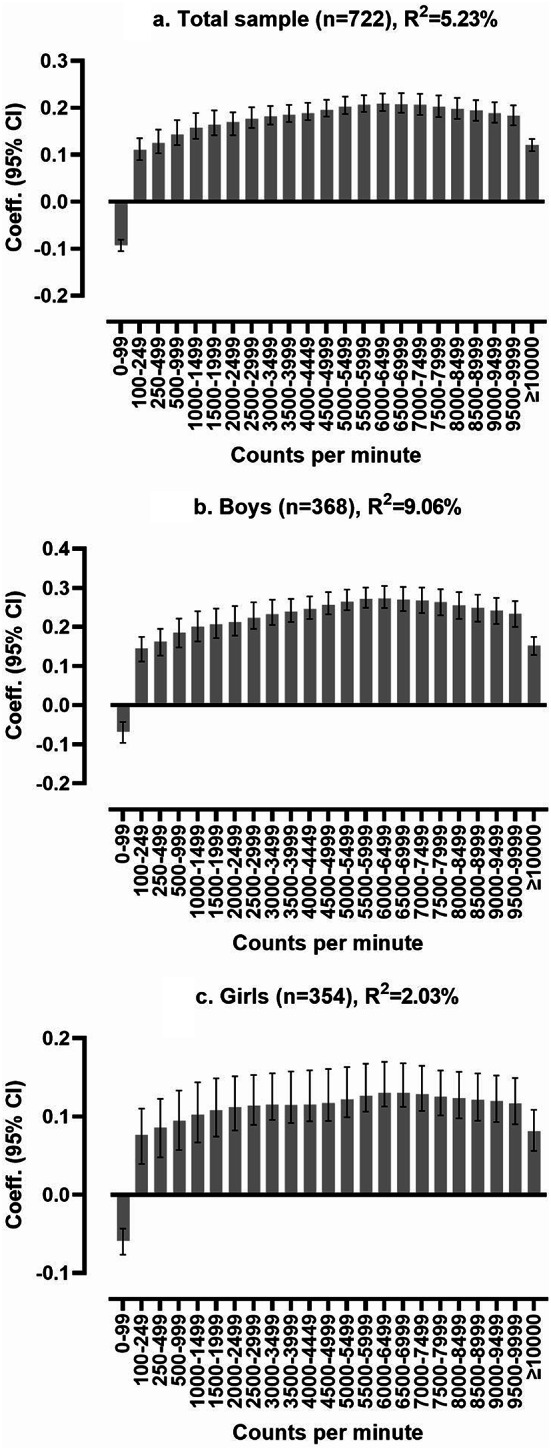
Partial least squares regression analysis of the multivariate physical activity signature associated with physical well-being, displayed as multivariate correlation coefficients. F1a. is adjusted for age, sex, and waist circumference, and F1b. and F1c. are adjusted for age and waist circumference. A positive bar indicates better physical well-being

An initial correlation analysis revealed that physical HRQoL was the only Kidscreen-27 dimension consistently associated with all four broad physical activity intensity levels (Suppl. Table 2). Using PLS-regression, these activity levels explained 4.15% of the variance in physical well-being. However, when we analyzed the 23 specific physical activity intensity levels, they accounted for a slightly higher percentage—5.23%—of the variation in physical well-being (Fig. 1a). Associations were negative for time spent in 0–99 cpm and positive for all intensities ≥ 100 cpm (all statistically significant). Associations between physical activity and physical well-being became stronger with increasing intensity levels up to approximately 6000–7000 cpm, after which they plateaued and diminished at even higher intensities.

**Fig. 2:**
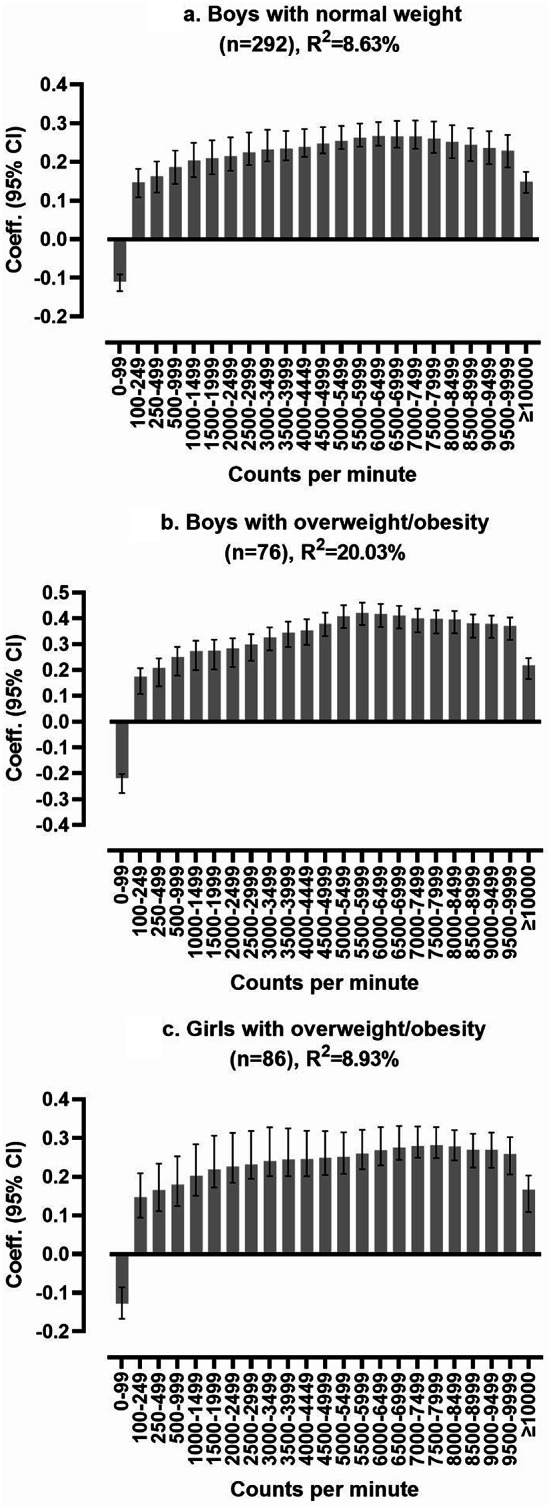
Stratified partial least squares regression analysis of the multivariate physical activity signatures associated with physical well-being, displayed as multivariate correlation coefficients. Results were adjusted for age. A positive bar indicates better physical well-being

The physical activity signature accounted for 2.03% and 9.06% of girls’ and boys’ physical well-being, respectively (Fig. 1b and c). For boys without overweight/obesity the explained variance was 8.63% (Fig. 2a), compared to 20.03% for those with overweight/obesity (Fig. 2b). We found no association in girls with normal weight (i.e., no predictive model allowing reporting of model fit or multivariate correlation coefficients). In contrast, the explained variance was 8.93% for girls with overweight/obesity (Fig. 2c). As for the total sample, higher intensity levels, approximately 6000–7000 cpm, exhibited the strongest association with physical well-being across all subsets.

## Discussion

Consistent associations were found between physical well-being and physical activity levels. High-intensity physical activity was especially associated with better physical well-being (except for girls with normal weight), and especially in children with overweight/obesity. Using more detailed physical activity intensity levels increased the explained variance in physical well-being.

We have previously investigated associations between physical activity and well-being in this sample [20]. However, these investigations relied only on the total cpm as an explanatory variable. To our knowledge, this is the first study to investigate multivariate physical activity profiles associated with well-being in children. Thus, a strength of this study is its novel insight into how an extensive range of physical activity intensity levels correlates with physical well-being. Multivariate pattern analyses can handle many strongly correlated physical activity intensity variables and thus allow for determining associations across the full intensity spectrum [9, 10], including more information from accelerometers than traditional analytic approaches. As demonstrated in this study, this approach may enhance the precision of the results. Other strengths include the relatively large sample size, balanced distribution of girls and boys, valid measurement tools, and analyses stratified by sex and overweight/obesity status.

Various mechanisms may explain how high-intensity physical activity positively influences physical well-being. Higher physical activity intensity may improve cardiovascular fitness, muscular strength, stress and worries, cognition, and a sense of mastery [26, 27]. Because these outcomes are more likely to result from physical activity rather than cause it, adjusting for them in studies like the current one could lead to overadjustment.

The sex disparities in this study raise questions about whether physical activity is more important for boys’ well-being than for girls’ well-being. Further, sex differences can be contextualized within the notion that boys and girls may possess divergent interests and motivations for physical activity [11, 28]. In this sample, girls may be preferer lower-intensity activities, leading to a weaker association between physical well-being and physical activity [11]. The findings may indicate the importance of differentiated physical activity approaches for boys and girls to promote enjoyable, high-intensity activities in both groups, as girls may prefer activities such as dancing, gymnastics, and jumping rope. In contrast, boys may prefer different ball sports and strength exercises [11].

An interesting finding was the pronounced associations between physical activity and physical well-being among children with overweight or obesity, particularly in boys. This aligns with previous studies indicating that physical activity can benefit vulnerable groups prone to health issues [26, 29]. It suggests that physical activity may be more critical for physical well-being in children who are overweight or obese, potentially because it enables them to participate in activities similarly to or together with children having a normal weight status. While this could engage those less inclined towards such activities, all forms of physical activity remain beneficial in addressing the effects of sedentariness. Facilitating a feeling of mastery in activities is essential for fostering healthy and sustained activity habits in the youth [30, 31]. In this study, we used waist circumference rather than BMI to classify overweight and obesity, which may be considered a strength. Approaches such as waist-to-height ratio or age- and sex-adjusted waist circumference, as applied here, may also capture adiposity more effectively and explain more variance in health outcomes [22, 32].

### Limitations

The study’s limitations are the cross-sectional design and the restricted age range of the participants. Moreover, although accelerometers are well-suited for measuring everyday physical activity, they cannot capture some activities, such as cycling and aquatic activities [33, 34]. As a result, children who frequently engage in these activities may appear to have lower activity levels than they actually do. This limitation could be partly addressed through self-report or proxy-report methods, although these approaches are also subject to recall bias and reduced accuracy. There are also multiple challenges with regard to data reduction of accelerometer physical activity data [35]. Thus, alternative approached could have affected our results, although most approaches probably would have little influence on relative physical activity levels within this sample and thus minimally affect our findings. Third, as PLS regression does not handle missing data, only participants with complete datasets were included in this study. However, previous sensitivity analyses in this sample suggest that ignoring missing data did not cause significant biases [20]. Finally, educational factors such as academic performance, parental education, and socioeconomic status may also influence both physical activity and HRQoL. While not included in our models, these factors could contribute to variation in outcomes and may be relevant to consider in future research.

## Conclusions

The findings indicate that while physical activity at all intensity levels was positively associated with physical well-being in children, the strongest effects were observed with high-intensity activity, particularly in boys and in children with overweight or obesity.

## Supplementary Information

Supplementary material 1.

## Data Availability

The dataset used in this paper is available from the EU Open Research Repository: https://zenodo.org/records/14002280.

## Abbreviations

ASK: Active Smarter Kids
Cpm: Counts per minute
HRQoL: Health-related quality of life
PLS: Partial least squares regression

## References

[1] The KIDSCREEN Group Europe. The KIDSCREEN questionnaires: quality of life questionnaires for children and adolescents (Handbook). Germany: Papst Science; 2006.

[2] Wu XY, Han LH, Zhang JH, Luo S, Hu JW, Sun K. The influence of physical activity, sedentary behavior on health-related quality of life among the general population of children and adolescents: A systematic review. PLoS ONE. 2017;12(11). 10.1371/journal.pone.0187668 PMC5679623 29121640

[3] Marker AM, Steele RG, Noser AE. Physical activity and health-related quality of life in children and adolescents: A systematic review and meta-analysis. Health Psychol. 2018;37(10):893. 30234348 10.1037/hea0000653

[4] Zhang T, Lu G, Wu XY. Associations between physical activity, sedentary behaviour and self-rated health among the general population of children and adolescents: a systematic review and meta-analysis. BMC Public Health. 2020;20:1–16. 32883275 10.1186/s12889-020-09447-1 PMC7650260

[5] Ávila-García M, Esojo-Rivas M, Villa-González E, Tercedor P, Huertas-Delgado FJ. Relationship between sedentary time, physical activity, and Health-Related quality of life in Spanish children. Int J Environ Res Public Health. 2021;18(5). 10.3390/ijerph18052702 PMC7967425 33800169

[6] Sales WB, Maranhão EF, Ramalho CST, Macêdo S, Souza GF, Maciel ÁCC. Early life circumstances and their impact on health in adulthood and later life: a systematic review. BMC Geriatr. 2024;24(1):978. 39609801 10.1186/s12877-024-05571-4 PMC11603629

[7] Aadland E, Kvalheim OM, Anderssen SA, Resaland GK, Andersen LB. The multivariate physical activity signature associated with metabolic health in children. Int J Behav Nutr Phys Activity. 2018;15(77). 10.1186/s12966-018-0707-z PMC6094580 30111365

[8] Aadland E, Andersen LB, Resaland GK, Kvalheim OM. Interpretation of multivariate association patterns between multicollinear physical activity accelerometry data and cardiometabolic health in Children-A tutorial. Metabolites. 2019;9(7):129. 31269708 10.3390/metabo9070129 PMC6680435

[9] Migueles JH, Aadland E, Andersen LB, Brønd JC, Chastin SF, Hansen BH, et al. GRANADA consensus on analytical approaches to assess associations with accelerometer-determined physical behaviours (physical activity, sedentary behaviour and sleep) in epidemiological studies. Br J Sports Med. 2022;56(7):376–84. 33846158 10.1136/bjsports-2020-103604 PMC8938657

[10] Craft BB, Carroll HA, Lustyk MK. Gender differences in exercise habits and quality of life reports: assessing the moderating effects of reasons for exercise. Int J Lib Arts Soc Sci. 2014;2(5):65–76. 27668243 PMC5033515

[11] Resaland GK, Aadland E, Andersen JR, Bartholomew JB, Anderssen SA, Moe VF. Physical activity preferences of 10-year‐old children and identified activities with positive and negative associations to cardiorespiratory fitness. 2019. 10.1111/apa.14487 29972701

[12] Reimers AK, Schoeppe S, Demetriou Y, Knapp G. Physical activity and outdoor play of children in public playgrounds—Do gender and social environment matter? Int J Environ Res Public Health. 2018;15(7). 10.3390/ijerph15071356 PMC6069007 29958386

[13] Kretschmer L, Salali GD, Andersen LB, Hallal PC, Northstone K, Sardinha LB, et al. Gender differences in the distribution of children’s physical activity: evidence from nine countries. Int J Behav Nutr Phys Activity. 2023;20(1):103. 10.1186/s12966-023-01496-0 PMC10478357 37667391

[14] King J, Jebeile H, Garnett S, Baur L, Paxton S, Gow M. Physical activity based pediatric obesity treatment, depression, self-esteem and body image: A systematic review with meta-analysis. Ment Health Phys Act. 2020;19:100342. 10.1111/ijpo.12600 32020780

[15] Resaland GK, Moe VF, Aadland E, Steene-Johannessen J, Glosvik Ø, Andersen JR, et al. Active smarter kids (ASK): rationale and design of a cluster-randomized controlled trial investigating the effects of daily physical activity on children’s academic performance and risk factors for non-communicable diseases. BMC Public Health. 2015;15(1):10. 26215478 10.1186/s12889-015-2049-y PMC4517398

[16] Resaland GK, Aadland E, Moe VF, Aadland KN, Skrede T, Stavnsbo M, et al. Effects of physical activity on schoolchildren’s academic performance: the active smarter kids (ASK) cluster-randomized controlled trial. Prev Med. 2016;91:7. 10.1016/j.ypmed.2016.09.005 27612574

[17] Resaland GK, Aadland E, Moe VF, Aadland KN, Skrede T, Stavnsbo M, et al. Effects of physical activity on schoolchildren’s academic performance: the active smarter kids (ASK) cluster-randomized controlled trial. Prev Med. 2016;91:322–8. 27612574 10.1016/j.ypmed.2016.09.005

[18] Association WM. WMA declaration of Helsinki - ethical prinsiples for medical research involving human subjects. 1964/2022. 19886379

[19] Andersen JR, Natvig GK, Haraldstad K, Skrede T, Aadland E, Resaland GK. Psychometric properties of the Norwegian version of the Kidscreen-27 questionnaire. Health Qual Life Outcomes. 2016;14(58):1–6. 27062022 10.1186/s12955-016-0460-4 PMC4826483

[20] Andersen JR, Natvig GK, Aadland E, Moe VF, Kolotkin RL, Anderssen SA, et al. Associations between health-related quality of life, cardiorespiratory fitness, muscle strength, physical activity and waist circumference in 10-year-old children: the ASK study. Qual Life Research: Int J Qual Life Aspects Treat Care Rehabilitation. 2017;26(12):3421–8. 10.1007/s11136-017-1634-1 28656535

[21] Evenson KR, Catellier DJ, Gill K, Ondrak KS, McMurray RG. Calibration of two objective measures of physical activity for children. J Sports Sci. 2008;26(14):1557–65. 18949660 10.1080/02640410802334196

[22] Brannsether B, Roelants M, Bjerknes R, Júlíusson PB. Waist circumference and waist-to-height ratio in Norwegian children 4–18 years of age: reference values and cut-off levels. Acta Pædiatrica. 2011;100(12):1576–82. 21627692 10.1111/j.1651-2227.2011.02370.x

[23] Rajalahti T, Kvalheim OM. Multivariate data analysis in pharmaceutics: A tutorial review. Int J Pharm. 2011;417(1):280–90. 21335075 10.1016/j.ijpharm.2011.02.019

[24] Wold S, Ruhe A, Wold H, Dunn WJ. The collinearity problem in linear regression. The partial least squares (PLS) approach to generalized inverses. SIAM J Sci Stat Comput. 1984;5(3):735–43.

[25] Kvalheim OM, Arneberg R, Grung B, Rajalahti T. Determination of optimum number of components in partial least squares regression from distributions of the root-mean-squared error obtained by Monte Carlo resampling. J Chemom. 2018;32(4):e2993.

[26] Mikkelsen K, Stojanovska L, Polenakovic M, Bosevski M, Apostolopoulos V. Exercise and mental health. Maturitas. 2017;106:48–56. 29150166 10.1016/j.maturitas.2017.09.003

[27] Belcher BR, Zink J, Azad A, Campbell CE, Chakravartti SP, Herting MM. The roles of physical activity, exercise, and fitness in promoting resilience during adolescence: effects on mental Well-Being and brain development. Biol Psychiatry Cogn Neurosci Neuroimaging. 2021;6(2):225–37. 33067166 10.1016/j.bpsc.2020.08.005 PMC7878276

[28] Fjørtoft I, Kjønniksen L, Støa EM. Barn - unge og fysisk aktivitet: Operasjonalisering av anbefalingene om fysisk aktivitet og stillesitting for barn og unge i alderen 0–18 år. Skriftserien fra Universitetet i Sørøst-Norge. 2018;12(69).

[29] Christiansen LB, Lund-Cramer P, Brondeel R, Smedegaard S, Holt A-D, Skovgaard T. Improving children’s physical self-perception through a school-based physical activity intervention: the move for Well-being in school study. Ment Health Phys Act. 2018;14:31–8.

[30] Deci EL, Ryan RM. Self-determination theory in health care and its relations to motivational interviewing: a few comments. Int J Behav Nutr Phys Activity. 2012;9(1):6. 10.1186/1479-5868-9-24 PMC3312850 22385839

[31] Ntoumanis N, Ng JYY, Prestwich A, Quested E, Hancox JE, Thøgersen-Ntoumani C, et al. A meta-analysis of self-determination theory-informed intervention studies in the health domain: effects on motivation, health behavior, physical, and psychological health. Health Psychol Rev. 2021;15(2):214–44. 31983293 10.1080/17437199.2020.1718529

[32] Brambilla P, Bedogni G, Heo M, Pietrobelli A. Waist circumference-to-height ratio predicts adiposity better than body mass index in children and adolescents. Int J Obes (Lond). 2013;37(7):943–6. 23478429 10.1038/ijo.2013.32

[33] Boyer WR, Wolff-Hughes DL, Bassett DR, Churilla JR, Fitzhugh EC. Accelerometer-Derived total activity counts, bouted minutes of moderate to vigorous activity, and insulin resistance: NHANES 2003–2006. Prev Chronic Dis. 2016;13:E146–E. 27763832 10.5888/pcd13.160159 PMC5072751

[34] Ridgers ND, Salmon J, Ridley K, O’Connell E, Arundell L, Timperio A. Agreement between activpal and actigraph for assessing children’s sedentary time. J Behav Nutr Phys Activity. 2012;9(15):8. 10.1186/1479-5868-9-15 PMC3311087 22340137

[35] Migueles JH, Cadenas-Sanchez C, Ekelund U, Delisle Nyström C, Mora-Gonzalez J, Löf M, et al. Accelerometer data collection and processing criteria to assess physical activity and other outcomes: A systematic review and practical considerations. Sports Med. 2017;47(9):1821–45. 28303543 10.1007/s40279-017-0716-0 PMC6231536

