# Supplementary material for: Linking 23 physical activity intensity levels to health-related quality of life in 10-year-old children
Source: BMC Res Notes. 2025 Oct 2;18:419. doi: 10.1186/s13104-025-07478-8 (PMC12492756; doi:10.1186/s13104-025-07478-8)
Supplement: Supplementary file 1 — Supplementary material 1. [file 13104_2025_7478_MOESM1_ESM.docx]

# Supplementary materials

Stai M, Aadland E, Andersen JR. Linking 23 physical activity intensity levels to health-related quality of life in 10-year-old children. 2024.

### Suppl. Table 1. Characteristics of boys and girls without and with overweight/obesity

| Variables,  mean ± sd | Boys without overweight/  Obesity (n=292) | Boys with overweight/ obesity (n=76) | Girls without overweight/  obesity (n=268) | Girls with overweight/ obesity (n=86) |
| --- | --- | --- | --- | --- |
| Age (year) | 10.2 ± 0.3 | 10.2 ± 0.3 | 10.2 ± 0.3 | 10.2 ± 0.3 |
| Waist circumference (cm) | 59.3 ± 6.7 | 73.6 ± 6.1 | 57.9 ± 3.8 | 72.2 ± 6.2 |
| Kidscreen-27 |  |  |  |  |
| Physical well-being | 53.5 ± 9.9 | 50.0 ± 10.1 | 51.4 ± 9.6 | 47.7 ± 8.3 |
| Psychological well-being | 53.5 ± 9.5 | 52.6 ± 10.5 | 54.1 ± 9.1 | 50.8 ± 7.7 |
| Autonomy & parents | 51.0 ± 9.8 | 48.8 ± 9.6 | 50.9 ± 9.2 | 50.6 ± 8.6 |
| Social support & peers | 51.7 ± 9.2 | 51.0 ± 11.1 | 51.7 ± 9.0 | 49.9 ± 8.8 |
| School environment | 53.3 ± 10.2 | 53.0 ± 10.5 | 55.9 ± 9.1 | 53.1 ± 8.5 |
| Physical activity |  |  |  |  |
| Counts per minute (min/day) | 770 ± 300 | 669 ± 282 | 685 ± 241 | 606 ± 238 |
| 0-99 | 593 ± 58 | 592 ± 63 | 597 ± 50.1 | 619 ± 58 |
| 100-249 | 17.9 ± 3.3 | 17.9 ± 2.9 | 18.1 ± 3.1 | 18.3 ± 2.8 |
| 250-499 | 21.1 ± 3.9 | 21.3 ± 3.5 | 21.0 ± 3.6 | 21.4 ± 3.4 |
| 500-999 | 30.6 ± 5.9 | 31.2 ± 5.4 | 29.8 ± 5.5 | 30.5 ± 5.3 |
| 1000-1499 | 22.8 ± 4.7 | 23.4 ± 4.5 | 21.7 ± 4.3 | 22.3 ± 4.2 |
| 1500-1999 | 20.6 ± 4.7 | 21.1 ± 4.3 | 19.4 ± 4.1 | 19.8 ± 4.1 |
| 2000-2499 | 15.3 ± 3.7 | 15.7 ± 3.5 | 14.1 ± 3.1 | 14.4 ± 3.3 |
| 2500-2999 | 12.9 ± 3.2 | 13.2 ± 3.2 | 11.7 ± 2.7 | 11.7 ± 3.0 |
| 3000-3499 | 11.9 ± 3.2 | 11.8 ± 3.3 | 10.6 ± 2.7 | 10.4 ± 2.9 |
| 3500-3999 | 8.5 ± 2.5 | 8.0 ± 2.7 | 7.4 ± 2.0 | 7.1 ± 2.2 |
| 4000-4449 | 7.0 ± 2.2 | 6.2 ± 2.2 | 5.9 ± 1.7 | 5.4 ± 1.8 |
| 4500-4999 | 6.4 ± 2.1 | 5.3 ± 1.8 | 5.3 ± 1.6 | 4.7 ± 1.6 |
| 5000-5499 | 4.7 ± 1.6 | 3.6 ± 1.2 | 3.8 ± 1.1 | 3.3 ± 1.2 |
| 5500-5999 | 3.9 ± 1.3 | 2.9 ± 0.9 | 3.1 ± 0.9 | 2.6 ± 1.0 |
| 6000-6499 | 3.6 ± 1.3 | 2.6 ± 0.9 | 2.8 ± 0.8 | 2.3 ± 0.9 |
| 6500-6999 | 2.6 ± 1.0 | 1.8 ± 0.7 | 2.0 ± 0.6 | 1.7 ± 0.6 |
| 7000-7499 | 2.1 ± 0.8 | 1.5 ± 0.6 | 1.7 ± 0.5 | 1.3 ± 0.5 |
| 7500-7999 | 1.9 ± 0.8 | 1.3 ± 0.6 | 1.5 ± 0.5 | 1.1 ± 0.5 |
| 8000-8499 | 1.4 ± 0.6 | 0.9 ± 0.4 | 1.1 ± 0.4 | 0.8 ± 0.3 |
| 8500-8999 | 1.1 ± 0.5 | 0.8 ± 0.3 | 0.9 ± 0.3 | 0.7 ± 0.3 |
| 9000-9499 | 1.0 ± 0.5 | 0.7 ± 0.3 | 0.8 ± 0.3 | 0.6 ± 0.3 |
| 9500-9999 | 0.8 ± 0.4 | 0.5 ± 0.2 | 0.6 ± 0.2 | 0.4 ± 0.2 |
| ≥10000 | 8.5 ± 7.6 | 6.1 ± 6.7 | 7.8 ± 5.8 | 6.0 ± 5.4 |

### Suppl. Table 2. Pearson correlations between the Kidscreen-27 and physical activity composite outcomes

| Variables | 2 | 3 | 4 | 5 | 6 | 7 | 8 | 9 | 10 |
| --- | --- | --- | --- | --- | --- | --- | --- | --- | --- |
| 1. Physical well-being | .52 | .41 | .35 | .38 | -.11 | .15 | .19 | .25 | .19 |
| 1. Psychological  well-being |  | .58 | .54 | .61 | -.06 | .07 | 0.05 | .07 | .04 |
| 1. Autonomy & parents |  |  | .48 | .45 | -.05 | -09 | .10 | .11 | .10 |
| 1. Social support & peers |  |  |  | .48 | -.11 | .08 | .05 | .07 | .09 |
| 1. School environment |  |  |  |  | -.04 | .02 | .01 | .04 | .04 |
| 1. Sedentary |  |  |  |  |  | -.24 | -.39 | -.39 | -.50 |
| 1. Ligh physical activity |  |  |  |  |  |  | .68 | .45 | .44 |
| 1. Moderate physical activity |  |  |  |  |  |  |  | .68 | .58 |
| 1. Vigorous physical activity |  |  |  |  |  |  |  |  | .90 |
| 1. Counts per minute |  |  |  |  |  |  |  |  |  |
